# POOR PHYSICAL FUNCTION EXPLAINS INCREASED WRIST FRACTURE RISK IN COMMUNITY DWELLING OBESE ICELANDIC ADULTS

**DOI:** 10.1093/geroni/igz038.607

**Published:** 2019-11-08

**Authors:** Alfons Ramel

**Affiliations:** University of Iceland, Reykjavik, Capital area, Iceland

## Abstract

Background: The associations between obesity, physical function, falls and fractures are unclear. Aim: The aim was to investigate whether obese adults have poorer sensory function, physical function as well as higher fracture risk than normal weight peers; and whether confounders explain potential differences between body mass index (BMI) categories. Methods: A case-control study was conducted using 98 wrist fracture cases (50-75 years) and 48 matched controls. Measurements included among others: anthropometrics, sensory function (Semmes-Weinstein-Monofilaments), physical function (Five-times-sit-to-stand test (FTSTS), questionnaires on previous fractures and fall history, the Activities-Specific-Balance-Confidence (ABC) and the Dizziness-Handicap-Inventory (DHI) scales. Results: Obese participants had lower physical function (FTSTS: +2.8sec,P<0.001), lower tactile sensitivity (monofilaments:+3.4 g,P=0.013), poorer ABC score (14.0,P<0.001), higher DHI score (11.6,P<0.001) and experienced more falls the previous 12 months (+0.8,P=0.006) compared to normal weight subjects. According to crude logistic regression analysis, the hazard ratios for wrist fracture in overweight and obese subjects were 2.7 (P=0.012) and 5.6 (P=0.002), respectively. When FTSTS was included in the statistical model (HR:1.7,P=0.004), it reduced the hazard ratios of overweight and obesity close to 1. Lifetime fractures (HR:5.5,P<0.001) and fall history (HR:4.3,P<0.001) were associated with an increased fracture risk independently from the BMI categories. Conclusion: Obese individuals have poorer physical function, higher risk of falls and increased wrist fracture risk compared to normal weight peers. Lower physical function is the main driver for the increased risk in obese subjects. Lifetime fractures and fall history are associated with an fracture independently from obesity.

